# Correction: Chow, J.W. and Stokic, D.S. Longitudinal Changes in Temporospatial Gait Characteristics during the First Year Post-Stroke. *Brain Sci.* 2021, *11*, 1648

**DOI:** 10.3390/brainsci12060715

**Published:** 2022-05-31

**Authors:** John W. Chow, Dobrivoje S. Stokic

**Affiliations:** Center for Neuroscience and Neurological Recovery, Methodist Rehabilitation Center, Jackson, MS 39216, USA; dstokic@mmrcrehab.org

The authors have made some changes in response to the reviewer’s comments after the manuscript was published in December 2021 [1]. The changes listed below are all new additions if not otherwise specified. Minor editorial changes are not included here.

## 1. General Changes

The unit for the gait speed has been changed from cm/s to m/s, and stride and step lengths and step width has been changed from cm to m.

## 2. Specific Changes

Abstract: Yet, only 5/24 (21%) of the participants improved speed from T1 to T2 according to speed-based minimum detectable change criteria.

Section 2.4. Statistical Analysis, Paragraph 2: To determine the speed outcome for each participant, we performed a Welch’s unequal variances t-test on the speeds of individual gait cycles in two consecutive evaluations [30,31] (see Appendix A for the derivation of gait cycle speed).

Section 2.4. Statistical Analysis, Paragraph 2: As an alternative to this approach, we also used the cut-offs for minimal detectable change (MDC) depending on the categories of walking speed (MDC = 0.10 m/s for the initial gait speed of <0.4 m/s; 0.15 m/s for 0.4–0.8 m/s; 0.18 m/s for >0.8 m/s) [32]. MDC is intended to distinguish true change from measurement errors.

Section 3. Results, Paragraph 6: We found a moderate correlation between the change in the FM-LE and the change in gait speed from T0 to T1 (*n* = 39, *r* = 0.42). However, such an association was not found from T1 to T2 (*n* = 22, *r* = 0.09). Consistent with the latter, both increases and decreases in FM-LE from T1 to T2 were observed in the 10 subjects who significantly increased in gait speed between the 6- and 12-month assessments.

Section 3. Results, Paragraph 7: Based on the speed-dependent MDC criteria, 72% (33/46) of the participants would be considered to have had true increases in gait speed from T0 to T1 and 5/24 (21%) from T1 to T2.

Section 4. Discussion: At the individual level, 76% of the participants significantly increased gait speed from 3–4 weeks to 6 months and 42% from 6 to 12 months post-stroke (72% and 21%, respectively, exceeded the MDC criteria).

Section 4.1. Longitudinal Changes, Paragraph 2: Though statistically non-significant, both the mean (online supplement, Table S1) and median (Figure 2) values indicate an overall change in the direction of improvement in most temporospatial parameters.

Section 4.1. Longitudinal Changes, Paragraph 4: To further address individual changes, we also used the MDC values for chronic stroke that take into account the baseline comfortable gait speed [32]. Accordingly, 72% of the participants increased gait speed from T0 to T1 and 21% from T1 to T2 to the degree that presumably exceeded the measurement error. However, caution should be exercised when applying MDC values across studies [32,42–46] since it is a “point estimate of the population value” [47–49] conditional upon the sample, measurement instrument, and settings. The same holds for the speed-dependent MDC cut-offs because of the known boundary effect, i.e., when the two individuals are just below and above the cut-off (e.g., 0.4 m/s), they are assigned different MDC values despite similar baseline speeds (e.g., MDC of 0.10 m/s for a baseline speed of 0.39 m/s vs. 0.15 m/s for 0.41 m/s, see Figure 2 in [32]).

Section 4.1. Longitudinal Changes, Paragraph 6: Regardless of the approach for determining individual changes in gait speed from 6 to 12 months post-stroke, the overall results indicate that gait speed can increase without corresponding changes in FM scores. Thus, compensation rather than recovery may underlie the increase in gait speed beyond 6 months post-stroke. Since at that time the majority of individuals neither improve gait speed nor typically receive therapy, future studies should identify interventions and candidates who may benefit from additional gait training.

Section 4.3. Study Limitations: The whole-body gait analysis was not performed to lessen the burden on the participants; however, to fully appreciate the reported temporospatial results, it would be informative to also have limb kinematic data.

Section 5. Conclusions: Among the individuals who can walk independently within 2 months post-stroke, the majority will significantly improve temporospatial gait measures during the first 6 months post-stroke followed by a plateau. Still, at least 20% and up to 40% may continue to increase gait speed from 6 to 12 months post-stroke.

The published version will be updated on the article webpage, with a reference to this correction notice.

## Supplementary Materials

The following supporting information can be downloaded at: https://www.mdpi.com/article/10.3390/brainsci12060715/s1, due to major changes and efforts in the revision of the original manuscript (10.3390/brainsci11121648), please see all details of the revised paper in the Supplementary Materials.
