# Supplementary material for: Longitudinal Changes in Temporospatial Gait Characteristics during the First Year Post-Stroke
Source: Brain Sci. 2021 Dec 15;11(12):1648. doi: 10.3390/brainsci11121648 (PMC8699066; doi:10.3390/brainsci11121648)
Supplement: Supplementary file 1 [file brainsci-11-01648-s001.zip › brainsci-1456638-supplementary.pdf]

SUPPLEMENT MATERIALS

For

Longitudinal Changes in Temporospatial Gait Characteristics During the First Year Post-Stroke

Table S1. Mean (SD) Fugl-Meyer Lower Extremity (FM-LE) Motor scores for the paretic leg and temporospatial gait parameters at the initial evaluation (T0) and at approximately 6 (T1) and 12 months (T2) post-stroke (*p*-values refer to paired t-tests).

	N=46				N=24			
	T0	T1	*p*-value	Cohen's *d*#	T1	T2	*p*-value	Cohen's *d*#
FM-LE Motor score	24.0 (5.9)	28.5 (5.4)	**<0.0001** [n=39]	-1.13	29.0 (5.3)	28.0 (4.9)	0.151 [n=22]	0.32
**Stride Parameter**								
Gait speed (cm/s)	56.2 (36.5)	88.9 (34.2)	**<0.0001**	-1.11	92.4 (34.2)	99.7 (30.9)	0.050	-0.42
Stride Length (cm)	85.4 (27.9)	108.3 (25.7)	**<0.0001**	-0.96	112.0 (26.9)	117.7 (24.4)	0.086	-0.37
Cadence (steps/min)	71.3 (28.5)	95.0 (21.2)	**0.0001**	-1.14	96.3 (19.0)	100.0 (17.3)	0.053	-0.42
Step width (cm)	13.6 (3.6)	13.8 (4.6)	0.652	-0.07	13.5 (4.0)	13.0 (3.3)	0.260	0.24
**Paretic Step Parameter**								
Stance time (%GC)	71.0 (7.9)	66.0 (4.3)	**0.0001**	0.64	65.6 (3.5)	65.0 (3.4)	0.226	0.25
Early double support time (%GC)	19.8 (6.7)	16.4 (3.4)	**0.0003**	0.58	16.3 (3.5)	15.9 (2.8)	0.484	0.15
Single support time (%GC)	25.2 (9.3)	31.6 (5.9)	**<0.0001**	-0.86	32.6 (4.7)	33.2 (4.1)	0.399	-0.18
Step length (cm)	44.5 (12.2)	55.2 (14.2)	**<0.0001**	-0.70	55.8 (12.8)	58.3 (12.2)	0.105	-0.35
Cadence (steps/min)	67.8 (29.9)	92.6 (23.4)	**<0.0001**	-1.21	94.8 (20.6)	98.6 (18.7)	0.065	-0.40
**Non-paretic Step Parameter**								
Stance time (%GC)	74.7 (9.5)	68.4 (5.9)	**<0.0001**	0.82	67.3 (4.7)	66.8 (4.1)	0.411	0.17
Early double support time (%GC)	26.0 (13.3)	18.1 (6.9)	**<0.0001**	0.70	16.8 (3.7)	15.8 (2.9)	0.106	0.34
Single support time (%GC)	28.9 (7.8)	33.9 (4.5)	**0.0001**	-0.64	34.2 (3.6)	35.1 (3.4)	0.143	-0.31
Step length (cm)	40.6 (16.9)	52.6 (15.1)	**<0.0001**	-0.87	55.7 (14.4)	58.9 (12.4)	0.077	-0.38
Cadence (steps/min)	78.0 (25.4)	98.6 (18.7)	**<0.0001**	-1.03	98.6 (18.3)	102.1 (16.4)	0.054	-0.42
**Symmetry Index (%)***								
Stance time	-4.8 (7.6)	-3.5 (6.7)	0.0367	0.32	-2.5 (7.1)	-2.8 (7.7)	0.472	-0.15
Early double support time	-19.6 (38.5)	-6.9 (18.7)	**<0.0001**	0.73	-3.4 (12.8)	0.4 (10.3)	0.053	0.42
Single support time	-17.8 (27.2)	-8.4 (17.1)	**0.0001**	0.64	-5.3 (16.0)	-5.9 (17.9)	0.331	-0.20
Step length	14.8 (35.7)	5.5 (30.4)	0.131	0.23	1.1 (10.4)	-1.1 (8.8)	0.944	0.01
Cadence	-18.6 (28.5)	-8.2 (16.9)	**<0.0001**	0.67	-4.6 (13.8)	-3.9 (13.6)	0.248	0.24

Significant differences in bold ($p \leq 0.0025$). GC: Gait cycle.
* Absolute values used in the *t*-tests.
# Effect size: small 0.2, medium 0.5, large 0.8 (Cohen, J. *Statistical Power and Analysis for the Behavior Sciences*, 2nd ed.; Lawrence Erlbaum Associates, Hillside, NJ, USA, 1988).
