# Supplementary material for: Correction: Chow, J.W. and Stokic, D.S. Longitudinal Changes in Temporospatial Gait Characteristics during the First Year Post-Stroke. Brain Sci. 2021, 11, 1648
Source: Brain Sci. 2022 May 31;12(6):715. doi: 10.3390/brainsci12060715 (PMC9221117; doi:10.3390/brainsci12060715)
Supplement: Supplementary file 1 [file brainsci-12-00715-s001.zip › brainsci-1696424-supplementary.pdf]

brain sciences

MDPI

Article

Longitudinal Changes in Temporospatial Gait Characteristics during the First Year Post-Stroke

John W. Chow * and Dobrivoje S. Stokic

Center for Neuroscience and Neurological Recovery, Methodist Rehabilitation Center, Jackson, MS 39216, USA; dstokic@mmrcrehab.org
* Correspondence: jchow@mmrcrehab.org

Abstract: Given the paucity of longitudinal data in gait recovery after stroke, we compared temporospatial gait characteristics of stroke patients during subacute (<2 months post-onset, T0) and at approximately 6 and 12 months post-onset (T1 and T2, respectively) and explored the relationship between gait characteristics at T0 and the changes in gait speed from T0 to T1. Forty-six participants were assessed at T0 and a subsample of twenty-four participants were assessed at T2. Outcome measures included Fugl-Meyer lower-extremity motor score, 14 temporospatial gait parameters, and symmetry indices of 5 step parameters. Except for step width, all temporospatial parameters improved from T0 to T1 ($p \leq 0.0001$). Additionally, significant improvements in symmetry were found for the initial double-support time and single-support time ($p \leq 0.0001$). As a group, no significant differences were found between T1 and T2 in any of the temporospatial measures. However, the individual analysis revealed that 42% (10/24) of the subsample showed a significant increase in gait speed (Welch's *t*-test, $p \leq 0.002$). Yet, only 5/24 (21%) of the participants improved speed from T1 to T2 according to speed-based minimum detectable change criteria. The increase in gait speed from T0 to T1 was negatively correlated with gait speed and stride length and positively correlated with the symmetry indices of stance and single-support times at T0 ($p \leq 0.002$). Temporospatial gait parameters and stance time symmetry improved over the first 6 months after stroke with an apparent plateau thereafter. A greater increase in gait speed during the first 6 months post-stroke is associated with initially slower walking, shorter stride length, and more pronounced asymmetry in stance and single-support times. The improvement in lower-extremity motor function and bilateral improvements in step parameters collectively suggest that gait changes over the first 6 months after stroke are likely due to a combination of neurological recovery, compensatory strategies, and physical therapy received during that time.

Keywords: gait speed; temporospatial parameters; subacute stroke; stroke recovery

Citation: Chow, J.W.; Stokic, D.S. Longitudinal Changes in Temporospatial Gait Characteristics During the First Year Post-Stroke. *Brain Sci.* **2021**, *11*, 1648. https://doi.org/10.3390/brainsci11121648

Academic Editor: Giovanni Morone

Received: 25 October 2021
Accepted: 13 December 2021
Published: 15 December 2021

Publisher's Note: MDPI stays neutral with regard to jurisdictional claims in published maps and institutional affiliations.

Copyright: © 2021 by the authors. Submitted for possible open access publication under the terms and conditions of the Creative Commons Attribution (CC BY) license (https://creativecommons.org/licenses/by/4.0/).

1. Introduction

Gait recovery after stroke has been a topic of interest for years. Marked improvements in gait have been demonstrated following conventional rehabilitation or specialized gait training in the first few months after stroke based on clinical scales such as the Functional Ambulatory Category or gait speed from a timed 10 m walk [1–6]. However, only a few studies investigated gait kinematics throughout recovery, and longitudinal data beyond 6 months post-stroke are scarce.

Within the first few months of stroke, cross-sectional studies revealed slower gait speed with reduced stride length and cadence in comparison to healthy adults [7–9]. Asymmetry in step parameters is also prominent, such as shorter stance, initial double-support and single-support times (as a percentage of the gait cycle time, %GC), and lower step cadence on the paretic side [8,9]. Gait speed is positively correlated with stride length,

cadence, and single-support time and negatively associated with stance and double-support times [7–9]. Gait speed is also significantly associated with select symmetry measures and clinically assessed motor impairments [7–9].

Longitudinal studies during the first few months of stroke reported significant increases in gait speed, stride length, cadence, step length, and single-support time [10–14]. The 5-meter speed within a week of stroke is positively correlated with the speed at 6 months post-stroke (r = 0.82) [15]. Studies that followed stroke participants up to a year post-stroke reported no improvement in clinical gait measures beyond the first few months [3,16–19], but changes in temporospatial gait parameters were not examined.

Temporospatial gait symmetry is believed to reflect gait quality [20]. Changes in gait symmetry during recovery were not as consistently found as changes in temporospatial gait parameters. Shin et al. [21] studied 6 men over 12 weeks of physical therapy starting within 1 month of stroke and reported a significant improvement in step length asymmetry but not step time asymmetry. Conversely, a decrease in swing time asymmetry but not step length asymmetry was reported in 61 participants from inpatient rehabilitation discharge (13–102 days post-stroke) to 6 months later [20]. Finally, significantly reduced asymmetry in both step length and step time was found after 10 weeks of gait training in 37 individuals within 1 month of stroke [13].

Given the paucity of longitudinal data, we examined here the changes in temporospatial gait parameters and Fugl-Meyer lower-extremity motor score (FM-LE) from within 2 months to 12 months post-stroke. We hypothesized significant changes from the initial evaluation within 2 months of a stroke to the 6-month evaluation (hypothesis 1) but not from the 6- to 12-month evaluation (hypothesis 2). Since gait speed is a reliable marker of ambulatory function after stroke but it is unknown whether early gait and motor impairments are related to the change in gait speed over time, we tested the hypothesis that initial temporospatial gait parameters and FM-LE would be associated with the change in gait speed at the 6-month evaluation (hypothesis 3). Because individual-level outcomes can provide additional insights into stroke recovery [18,22], we examined in each participant the changes in gait speed between the initial and 6-month evaluations, as well as between the 6- and 12-month evaluations.

2. Materials and Methods

2.1. Participants

The inclusion criteria for this study were first documented stroke in the preceding 2 months, able to follow simple instructions, able to walk independently for 7 m with or without assistive devices, and no artificial lower-extremity joint replacement. After completing the initial evaluation shortly before discharge from inpatient rehabilitation (T0), the participants were invited to return for a follow-up at approximately 6 months (T1) and 12 months (T2) post-stroke. All participants signed the informed consent form approved by the institutional review board (IRB) and received the IRB-approved travel allowance for each follow-up visit.

Prior to gait evaluation, the paretic limb was assessed using the FM-LE motor section [23,24] (range 0–34, not performed on a few occasions when the research therapist was not available). Muscle hypertonia was assessed in 10 muscle groups around the hip, knee, and ankle joints on the paretic side using the modified Ashworth scale (range 0–5) [25].

2.2. Gait Evaluation

Because of changes in lab protocol, gait data were collected from 31 subjects with a GAITRite (4.3 m in length, CIR Systems, Inc., Franklin, NJ, USA) or Zeno (6.1 m in length, ProtoKinetics LLC, Havertown, PA, USA) electronic walkway. In 15 subjects, gait data were collected over an 8 m hard surface walkway using 12 digital cameras operated at 60 Hz (Motion Analysis Corp., Rohnert Park, CA, USA), 5 force plates sampled at 1200 Hz (Type 4060; Bertec Corp., Columbus, OH, USA), and the Helen Hayes marker system [26].

Participants were asked to walk back and forth along the walkway 4–5 passes in their shoes at a self-selected comfortable free speed and with a customary assistive device, if any (Table 1). Use of a short, non-rigid polypropylene ankle-foot orthosis on the paretic side was allowed to prevent foot drop. The agreement of temporospatial parameters between the electronic walkway and motion capture system has been established for stroke participants [27].

Table 1. Subject demographics, Fugl-Meyer scores, and average (SD) gait speeds at initial evaluation (T0) and at approximately 6 (T1) and 12 months (T2) post-stroke along with changes in gait speed. Bold IDs indicate hemorrhagic strokes (all others ischemic). If used, the assistive device is indicated next to speed (see footnotes).

ID	Sex	Age [year]	BMI	Onset to T0 [Days]	Paretic Side	Paretic FM-LE T0	T1	T2	Gait Speed [m/s] T0	T1	T2	Speed Change [m/s] T1-T0 *	T2-T1 #
1	M	74	27	16	L	na	33	–	0.20 (0.03) [W]	0.85 (0.05) [C]	–	**0.65**	–
2	M	74	26	16	L	na	24	27	0.37 (0.03) [W]	0.36 (0.05) [W]	0.63 (0.05) [C]	−0.01	**0.27**
3	M	82	23	12	L	30	34	–	0.79 (0.05)	0.87 (0.04)	–	0.08	–
4	M	65	26	13	L	32	34	–	0.72 (0.06)	1.28 (0.07)	–	**0.56**	–
5	F	54	25	14	L	24	27	–	0.59 (0.07) [C]	0.78 (0.06) [C]	–	**0.19**	–
6	M	59	28	22	L	16	22	–	0.7 (0.02) [OW]	0.64 (0.03) [OC]	–	**0.57**	–
7	M	72	36	11	L	31	32	32	0.63 (0.07) [W]	0.85 (0.07)	0.91 (0.04)	**0.22**	0.06
**8**	M	73	35	20	R	32	na	34	0.78 (0.05)	1.13 (0.06)	1.32 (0.04)	**0.35**	**0.19**
9	F	63	36	25	R	28	31	–	0.20 (0.02) [C]	0.16 (0.02) [W]	–	−0.04	–
10	M	42	30	15	L	18	33	27	0.46 (0.04) [C]	1.51 (0.03)	1.51 (0.06)	**1.05**	0.00
11	M	55	26	17	R	16	19	21	0.16 (0.02) [C]	0.50 (0.02) [C]	0.52 (0.02) [C]	**0.34**	0.02
12	F	70	30	21	R	23	31	–	0.17 (0.02)	0.76 (0.09)	–	**0.59**	–
13	F	39	32	14	L	28	29	–	0.36 (0.02)	1.24 (0.10)	–	**0.88**	–
14	M	49	32	12	R	14	20	–	0.12 (0.01) [OC]	0.37 (0.03) [OC]	–	**0.25**	–
15	F	61	23	22	L	30	34	34	1.07 (0.05)	1.40 (0.10)	1.41 (0.09)	**0.33**	0.01
16	F	48	38	29	L	14	24	–	0.16 (0.03) [C]	1.02 (0.13) [C]	–	**0.86**	–
17	M	49	33	11	R	17	33	30	0.28 (0.04)	1.16 (0.09)	1.04 (0.08)	**0.88**	**−0.12**
18	M	67	26	24	R	27	na	–	0.19 (0.02) [C]	0.25 (0.04) [C]	–	**0.06**	–
19	F	73	24	15	L	30	34	32	0.60 (0.07) [C]	0.94 (0.12) [C]	0.92 (0.06)	**0.34**	−0.02
20	M	65	29	12	L	33	34	30	1.02 (0.03)	0.94 (0.07)	1.02 (0.03)	−0.08	**0.08**
21	M	48	33	15	L	23	29	–	0.48 (0.03)	0.87 (0.04)	–	**0.39**	–
22	M	76	28	9	L	24	34	31	1.23 (0.03)	1.29 (0.09)	1.20 (0.10)	0.06	−0.09
**23**	M	56	25	38	L	23	na	na	0.36 (0.04) [OC]	0.49 (0.04) [OC]	0.41 (0.02) [OC]	**0.13**	−0.08
24	M	57	28	60	L	21	32	32	0.26 (0.02)	0.44 (0.08)	1.14 (0.08)	**0.18**	**0.70**
25	F	75	21	12	R	29	32	–	0.48 (0.03) [W]	1.23 (0.07)	–	**0.75**	–
26	F	75	23	38	L	33	34	–	1.33 (0.09)	1.37 (0.05)	–	0.04	–
27	F	57	31	52	R	25	29	25	0.85 (0.04)	0.94 (0.08)	0.91 (0.06) [O]	**0.09**	−0.03
28	F	44	33	48	R	23	29	–	0.73 (0.05)	0.99 (0.05)	–	**0.26**	–
29	F	32	33	31	L	26	28	–	0.54 (0.03)	0.77 (0.04)	–	**0.23**	–
**30**	M	53	29	45	R	11	18	22	0.16 (0.01) [W]	0.41 (0.03) [C]	0.45 (0.02) [C]	**0.25**	**0.04**
31	M	63	27	35	L	24	28	–	0.96 (0.07) [C]	0.93 (0.04)	–	−0.03	–
32	F	61	35	42	L	25	32	29	0.85 (0.03)	0.83 (0.09)	0.89 (0.08)	−0.02	0.06
33	F	51	22	17	R	26	na	–	1.09 (0.06)	1.29 (0.012)	–	**0.20**	–
**34**	F	54	33	34	L	27	27	–	0.56 (0.10)	0.59 (0.012) [C]	–	0.03	–
35	F	67	41	17	L	29	34	27	0.79 (0.07)	0.87 (0.10)	1.07 (0.09)	0.08	**0.20**
36	M	48	32	41	R	24	25	29	0.26 (0.03) [OC]	0.85 (0.03)	0.96 (0.06) [O]	**0.59**	**0.11**
37	F	61	24	42	L	25	32	29	0.15 (0.02)	0.83 (0.08)	0.89 (0.08)	**0.68**	0.06
38	M	57	26	32	R	18	18	12	0.15 (0.02) [OC]	0.48 (0.03) [OC]	0.65 (0.05) [O]	**0.33**	**0.17**

39	M	66	30	16	L	28	30	33	1.15 (0.17)	1.19 (0.08)	1.13 (0.10)	0.04	−0.06
40	M	57	25	28	R	30	30	30	1.11 (0.10)	1.39 (0.03)	1.54 (0.06)	**0.28**	**0.15**
41	F	55	33	14	R	21	27	–	0.33 (0.01)	1.18 (0.10)	–	**0.85**	–
42	F	49	34	53	R	na	25	25	0.83 (0.06)	1.12 (0.15)	1.02 (0.09)	**0.29**	−0.10
43	F	51	22	14	R	26	29	29	1.09 (0.06)	1.29 (0.12)	1.23 (0.11)	**0.20**	−0.06
44	M	71	28	22	L	28	27	29	0.74 (0.06)	0.93 (0.10)	1.10 (0.04)	**0.19**	**0.17**
45	M	64	27	22	R	19	22	–	0.20 (0.02) [C]	0.86 (0.07) [C]	–	**0.66**	–
46	F	65	27	33	L	13	13	–	0.15 (0.02) [W]	0.38 (0.04) [C]	–	**0.23**	–
Mean		59.7	29.0	25.0		24.3	28.4	28.2	0.56	0.89	1.00	0.33	0.07
SD		11.1	4.7	13.3		5.8	5.3	4.9	0.36	0.24	0.31	0.30	0.17

Significant speed change in bold (Welsch's t-test): * from T0 to T1 ($p \leq 0.0011$); [#] from T1 to T2 ($p \leq 0.0021$). Negative change in speed (i.e., a decrease in speed) in red. Assistive device use: [O] ankle-foot orthosis, [C] cane, and [W] walker. BMI: body mass index; FM-LE: Fugl-Meyer lower-extremity motor score; na: not available; M: male; F: female; L: left; R: right.

2.3. Data Reduction

For data collected using an electronic walkway, GAITRite (CIR Systems, Inc.) or PKMAS (ProtoKinetics LLC) software was used to export the toe and heel locations and timing of each initial foot contact and toe-off. For data collected by a motion capture system, OrthoTrak Gait Analysis software (Motion Analysis Corp) was used to process marker location data and to determine footfall instants based on a combination of ground reaction force and foot kinematics [28]. Only strides that occurred in the mid-section of the walkway were included in subsequent analyses and performed using a custom program written in MATLAB (The MathWorks, Inc., Natick, MA, USA). As a result, 10–25 gait cycles were analyzed for each participant at each time point.

A gait cycle (GC) was defined by two consecutive initial foot contacts of the same foot. Only full gait cycles were analyzed based on the presence of all 5 critical instants (first ipsilateral initial foot contact, contralateral toe-off, contralateral initial foot contact, ipsilateral toe-off, and next ipsilateral initial foot contact). The 14 analyzed temporospatial parameters were gait speed, stride length, stride cadence, step width, paretic and non-paretic stance time (%GC), early double-support time (%GC), single-support time (%GC), step length, and step cadence.

Temporospatial symmetry was assessed with a symmetry index {SI = (paretic − non-paretic)/[0.5 × (paretic + non-paretic)] × 100%} for each of the 5 step parameters [8]. This computation of SI is preferred for descriptive purposes because it indicates both the direction and magnitude of the asymmetry. However, absolute SI values were used in statistical analysis to emphasize the magnitude of deviation from ideal symmetry (i.e., SI = 0) regardless of direction [29]. This way, changes in SI from 10% to −5% and from 10% to 5% are considered equal improvements (i.e., trending toward zero SI).

2.4. Statistical Analysis

Mean and SD were calculated for FM-LE and 19 temporospatial/SI parameters at each of the 3 evaluation points. For the first two hypotheses, these outcome measures were compared between T0 and T1 (hypothesis 1, N = 46) and between T1 and T2 (hypothesis 2, N = 24) using a two-tailed paired *t*-test. The third hypothesis was tested by deriving coefficients of correlation between the change in gait speed from T0 to T1 and each outcome at T0. Because the included variables were not normally distributed (skewness > ±1), the Spearman rank correlation was calculated. To adjust for the total of 20 outcomes, the level of significance was set at $\alpha = 0.0025$ (0.05/20) for the paired *t*-tests and correlations.

To determine the speed outcome for each participant, we performed a Welch's unequal variances *t*-test on speeds of individual gait cycles at two consecutive evaluations [30,31] (see Appendix A for the derivation of gait cycle speed). Given the sample size of 46 at T1 and 24 at T2, the Bonferroni-adjusted alpha levels for the comparisons between T0 and T1 and T1 and T2 were 0.0011 (0.05/46) and 0.0021 (0.05/24), respectively. In case

of a significant change in speed, the sign (positive or negative) was used to designate speed *increase* or *decrease*. A non-significant Welch's *t*-test was taken as *no change* in speed. As an alternative to this approach, we also used the cut-offs for minimal detectable change (MDC) depending on the categories of walking speed (MDC = 0.10 m/s for the initial gait speed of <0.4 m/s; 0.15 m/s for 0.4-0.8 m/s; 0.18 m/s for >0.8 m/s) [32]. MDC is intended to distinguish true change from measurement error.

3. Results

Of the 46 participants examined initially (T0) and at the first follow-up (T1), 24 returned for the second follow-up (T2). The T0 evaluation occurred at 25 ± 13 days post-stroke (range 9–60 days, median 21.5, interquartile range 14–34), T1 at 6.6 ± 0.7 months post-stroke (range 5.5–8.2 months), and T2 at 12.7 ± 0.4 months post-stroke (12.0–13.7 months). The baseline characteristics of the participants that returned for the second follow-up (n = 24) were not significantly different from those that were lost (n = 22) in terms of age (58 ± 13 vs. 61 ± 10 years, p = 0.623, 2-tailed unpaired *t*-test), time post-stroke (23 ± 10 vs. 27 ± 16 days, p = 0.267), FM-LE (24 ± 6 vs. 25 ± 6, p = 0.610), and gait speed both at T0 (0.48 ± 0.35 vs. 0.64 ± 0.37 m/s, p = 0.128) and T1 (0.85 ± 0.35 vs. 0.92 ± 0.34 m/s, p = 0.467).

Based on self-report, all participants received outpatient physical and/or occupational therapy between T0 and T1 during the first 2–3 months post-discharge (2–3 times per week). Only one participant reported receiving regular physical therapy between T1 and T2 (Table 1, #44). They received outpatient therapy at different clinics throughout the state, in which the content of the therapy was not possible to monitor prospectively or retrieve retrospectively.

Table 1 shows demographic, clinical, and gait speed data along with the use of an ankle-foot orthosis (AFO) and walking aids (if any) at each time point. The changes in gait speed between the consecutive evaluations are also given. Aside from the Ashworth score of 1 in 6 of 460 (1.3%) paretic muscle groups at T0 (1 group in 4 participants, 2 groups in 1 participant), all other scores were 0, including at T1 and T2. Compared to those who did not use an assistive device at T0 (n = 29), the users of an AFO and/or walking aids (n = 17) had lower FM-LE (21.4 ± 6.0 vs. 25.8 ± 5.1, p = 0.015 2-tailed unpaired *t*-test) and gait speed (0.42 ± 0.30 vs. 0.64 ± 0.38 m/s, p = 0.044).

In terms of the changes from T0 to T1 (first hypothesis), both the FM-LE motor score and gait speed significantly increased, the latter being due to significant increases in both stride length and cadence (Figure 1). Bilaterally decreased stance and early double-support time and increased single-support time, step length, and step cadence were also found. Significant improvements in symmetry (SI trending towards zero) were detected in the early double-support and single-support times and in step cadence. The effect size of the reported significant differences between T0 and T1 falls in the moderate to large range (0.58 to 1.21). As for the changes from T1 to T2 (hypothesis 2), no significant differences were found (Figure 2). Means and SDs for all outcome measures at different time points and corresponding p- and Cohen's d values are given in the online supplement (Table S1).

Regarding the third hypothesis, the change in gait speed from T0 to T1 correlated negatively with the initial (T0) gait speed, stride length, paretic single-support time, step length, step cadence, and non-paretic step length and positively with the non-paretic stance time and SIs of stance and single-support times (Figure 3). All significant correlations were moderate ($|r|$ = 0.44–0.55) [33,34].

Figure 4 shows gait speed trajectories for individual participants. According to the Welch's *t*-test, 35 out of 46 (76%) participants increased gait speed from T0 to T1, whereas 10 (22%) showed no change, and 1 (2%) decreased gait speed (Table 1). The initial FM-LE was lower for the group that increased speed from T0 to T1 (23.1 ± 5.9, n = 33) compared with the group that did not increase the speed (28.1 ± 3.3, n = 10). From T1 to T2, 10 of 24 (42%) participants increased the speed, and 2 (8%) decreased the speed. Among the 10 who increased the speed between T1 and T2, 7 also did so between T0 and T1, whereas 3

did not. We found a moderate correlation between the change in the FM-LE and the change in gait speed from T0 to T1 (n = 39, r = 0.42). However, such association was not found from T1 to T2 (n = 22, r = 0.09). Consistent with the latter, both increases and decreases in FM-LE from T1 to T2 were observed in the 10 subjects who significantly increased in gait speed between the 6- and 12-month assessments.

Based on the speed-dependent MDC criteria, 72% (33/46) of the participants would be considered to have true increase in gait speed from T0 to T1 and 5 of 24 (21%) from T1 to T2.

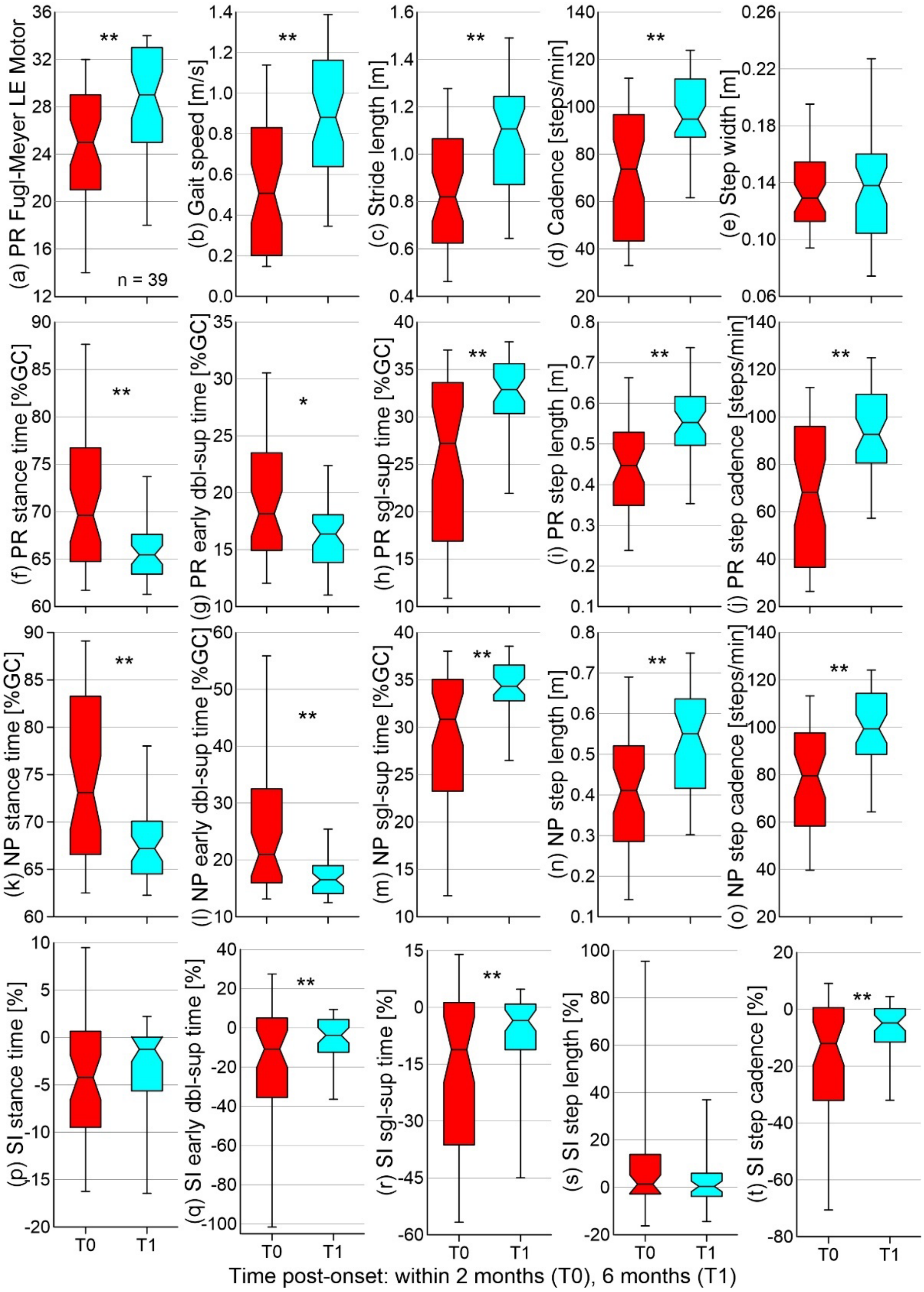

Figure 1. Box-and-whisker plots for the (**a**) Fugl-Meyer lower-extremity motor score, (**b**–**o**) temporospatial gait parameters, and (**p**–**t**) symmetry indices collected in 46 participants at baseline (T0) and approximately 6 months post-stroke (T1).

Lines indicate the median value and the first and third quartiles, whereas the whiskers are the 5th and 95th percentiles. The notch displays the 95% confidence interval around the median. Asterisks indicate significant differences between T0 and T1 at $p \leq 0.0025$ (*) or $p \leq 0.0001$ (**). A negative symmetry index indicates a larger value on the non-paretic side. PR: paretic; NP: non-paretic; SI: symmetry index; dbl-sup: double-support; and sgl-sup: single-support.

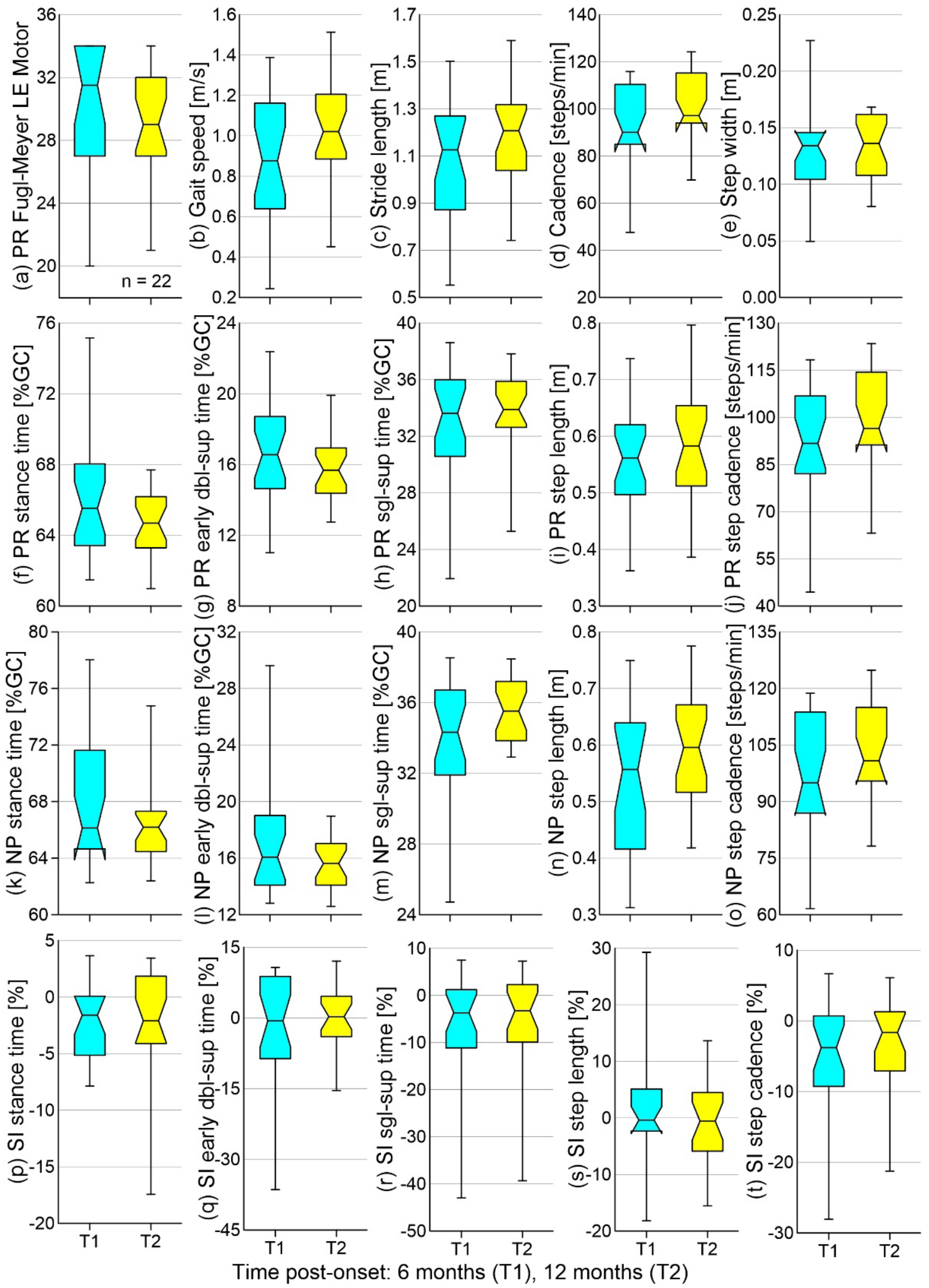

Figure 2. Box-and-whisker plots for the (**a**) Fugl-Meyer lower-extremity motor score, (**b**–**o**) temporospatial gait parameters, and (**p**–**t**) symmetry indices collected in 24 participants at approximately 6 months (T1) and 12 months (T2) post-stroke (line, whisker, and notch designations as in Figure 1). A negative symmetry index indicates a larger value on the non-paretic side. PR: paretic; NP: non-paretic; SI: symmetry index; dbl-sup: double-support; and sgl-sup: single-support.

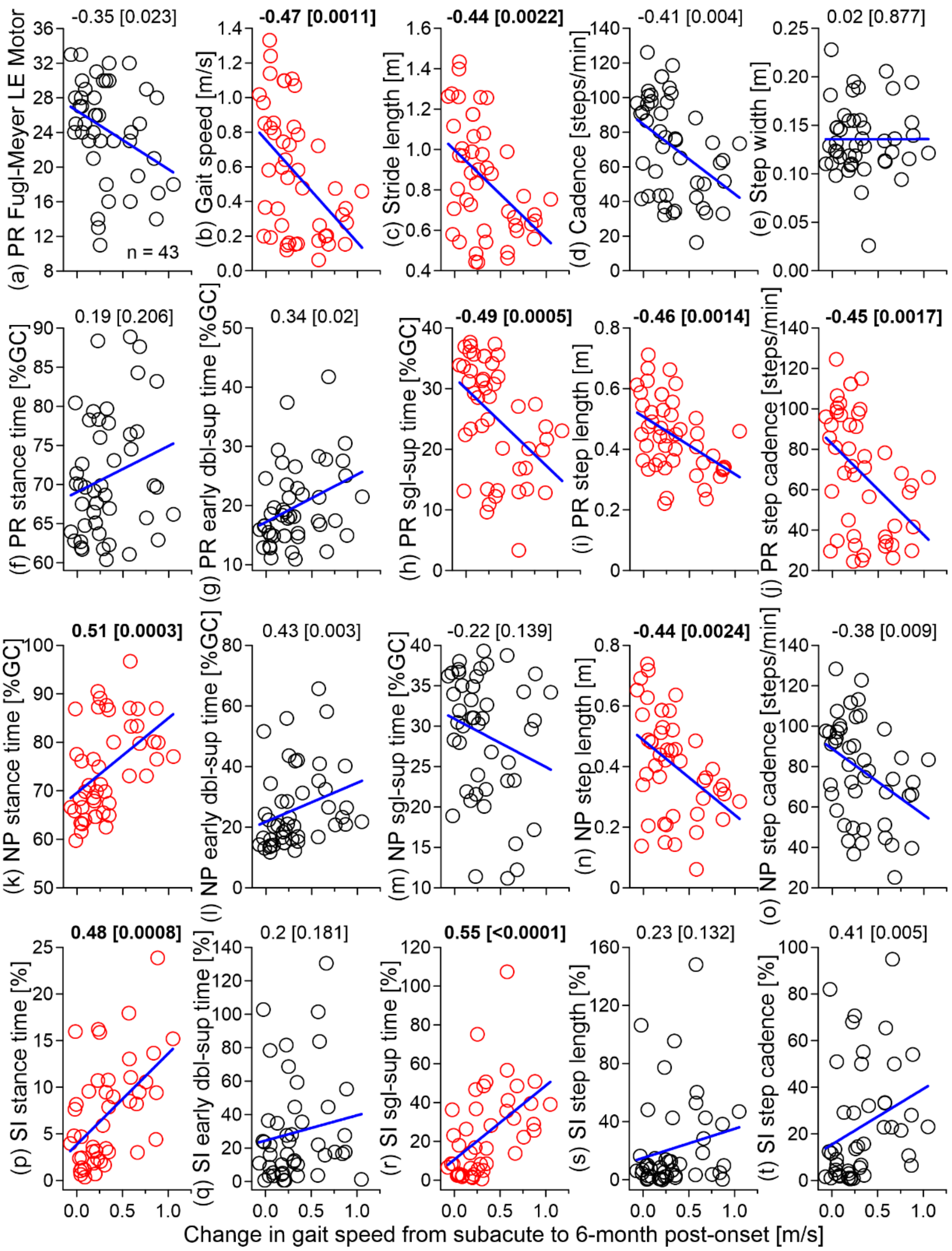

Figure 3. Spearman correlations between (**a**) Fugl-Meyer lower-extremity motor score, (**b**–**o**) temporospatial gait parameters, and (**p**–**t**) absolute values of symmetry indices at T0 and the change in gait speed from T0 to T1. The associated P-values are in parentheses and significant correlations ($p \leq 0.0025$) are in bold. PR: paretic; NP: non-paretic; SI: symmetry index; dbl-sup: double-support; and sgl-sup: single-support.

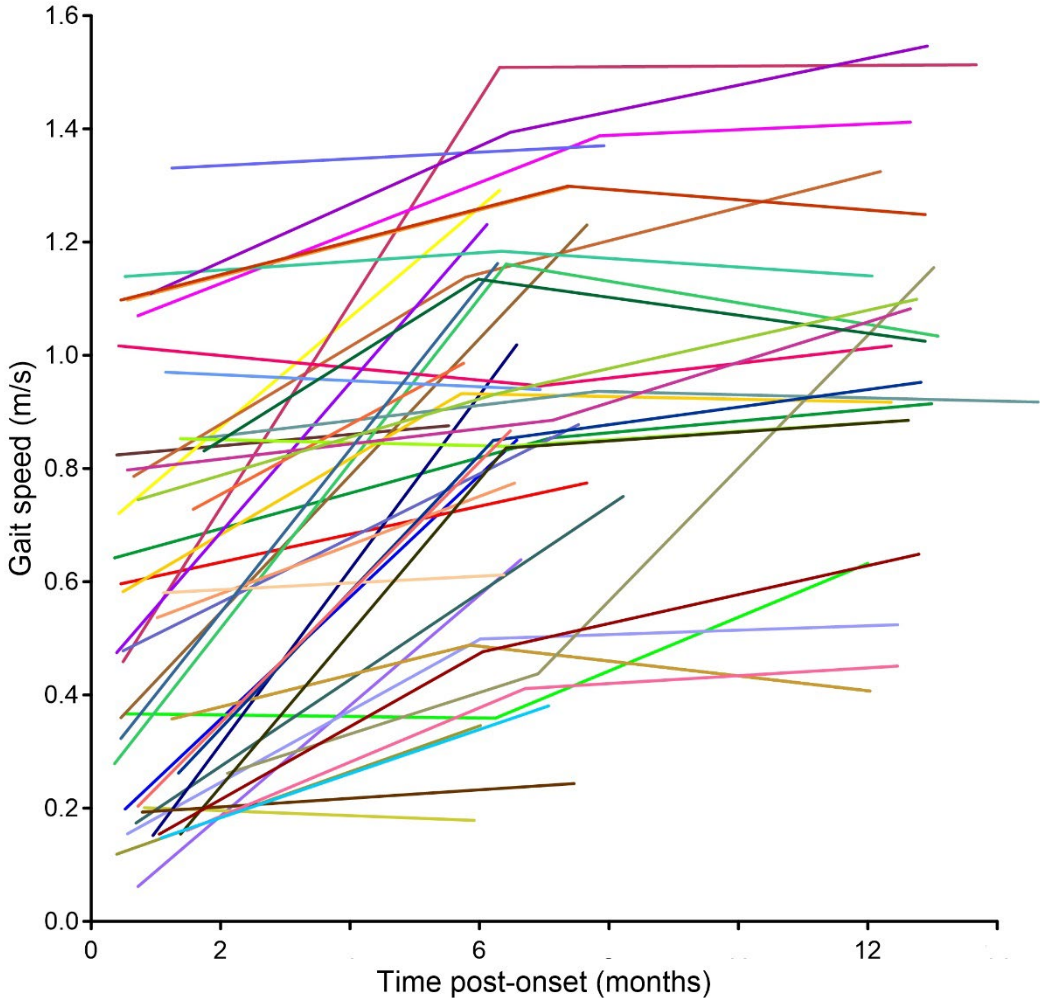

Figure 4. Trajectories of gait speeds of individual participants across T0, T1, and T2.

4. Discussion

The results of this study revealed significant improvements in residual motor function of the paretic leg and temporospatial gait characteristics from 3–4 weeks to 6 months after stroke. However, no significant changes were observed between 6 and 12 months post-stroke. At the individual level, 76% of the participants significantly increased gait speed from 3–4 weeks to 6 months and 42% from 6 to 12 months post-stroke (72% and 21%, respectively, exceeded the MDC criteria) . The change in gait speed at 6 months post-stroke was moderately associated with several temporospatial and symmetry measures but not with the initial motor impairment of the paretic leg. As a group, however, individuals who significantly increased the speed had greater initial motor impairment.

We recruited participants from a pool of consecutive stroke admissions and close to the time of discharge from inpatient rehabilitation, which is typically completed within 2 months post-stroke in the United States. Compared to other longitudinal studies, the average gait speed of our sample at 3–4 weeks post-stroke (0.56 ± 0.36 m/s) is comparable to the participants in Alingh et al. [10] (0.54 ± 0.36 m/s, N = 32, < 10 week-post), but slower than those in Duncan et al. [35] (0.65 ± 0.29 m/s, N = 92, 76 ± 28 day-post), Rozanski et al. [20] (0.88 ± 0.32 m/s, N = 61, 44 ± 20 days post-stroke), and Aaslund et al. [15] (0.95 ± 0.31 m/s, N = 101, 5 ± 2 days post-stroke). Participants' initial gait function needs to be related to the time post-stroke and access to therapy when comparing results from different longitudinal studies because the changes over time are influenced by several factors.

4.1. Longitudinal Changes

Overall, hypothesis 1 seems supported since significant differences between the initial and 6-month evaluations were found for the paretic leg motor impairment in the paretic leg (FM-LE) and almost all studied temporospatial gait parameters (17/19). These results agree with previous reports of significant improvements in gait kinematics during the first 6 months of stroke [10–12,21]. However, there are some discrepancies. For example, contrary to the previous reports [10,11,21], we did not find significant changes in step width and step length symmetry. The absence of a significant difference in the mean SI of the step length at T0 and T1 could have been due to the large sample variance in absolute SI values at the two evaluation points (range 0 to 148% at T0 and 0.2% to 140% at T1). The bilateral improvements in step parameters suggest that the compensation by the non-paretic side was not the main contributor to the overall faster gait speed at 6 months post-stroke. Further evidence of improvements specific to the paretic leg is the reduced asymmetry during the initial loading and single-support phases (SI values of early double-support time and single-support time trending toward zero).

The lack of significant differences between 6 and 12 months post-stroke support hypothesis 2. Though statistically non-significant, both the mean (online supplement, Table S1) and median (Figure 2) values indicate an overall change in the direction of improvement in most temporospatial parameters. In aggregate, our results agree with other longitudinal studies reporting that the major recovery of motor function takes place within the first few months of stroke [36–41]. Less prominent improvements in gait past the first 6 months may be due to a decreased capacity for neuroplasticity later in the course of recovery or no provision/less intense outpatient therapy between 6 and 12 months post-stroke.

At the individual level, however, we did observe different changes in gait speed over time (Figure 4) that were further analyzed first statistically (Welch's *t*-test). Approximately 75% of the initial sample improved gait speed at 6 months post-stroke and so did 40% of the available sample at 12 months post-stroke. Among the latter, most (7/10) showed steady improvements throughout the first 12 months, whereas the remaining 3 (Table 1, #2/#20/#35) showed no early but late improvement only.

To futher address individual changes, we also used the MDC values for chronic stroke that take into account the baseline comfortable gait speed [48]. Accordingly, 72% of the participants increased gait speed from T0 to T1 and 21% from T1 to T2 to a degree that presumably exceeded the measurement error. However, caution should be exercised when applying MDC values across studies [32,42–46] since it is a "point estimate of the population value" [47–49] conditional upon the sample, measurement instrument, and settings. The same holds for the speed-dependent MDC cut-offs because of the known boundary effect; i.e., when the two individuals are just below and above the cut-off (e.g., 0.4 m/s), they are assigned different MDC values despite similar baseline speeds (e.g., MDC of 0.10 m/s for a baseline speed of 0.39 m/s vs. 0.15 m/s for 0.41 m/s; see Figure 2 in [32]).

In terms of the three ambulation classes based on gait speed proposed by Duncan et al. [1] and Schmid et al. [50], 20 (43%) of our participants were household ambulators (<0.4 m/s) at 3–4 weeks post-stroke, and 15 of them (75%) progressed to limited community ambulators (0.4–0.8 m/s) at 6 months post-stroke. Similarly, 10 (71%) of the 14 initially limited community ambulators became full community ambulators (>0.8 m/s) at 6 months post-stroke. Overall, our findings highlight the need to go beyond group analysis and focus on individual trajectories of recovery when evaluating the progress and outcomes of stroke rehabilitation [18,22].

Regardless of the approach for determining individual changes in gait speed from 6 to 12 months post-stroke, the overall results indicate that gait speed can increase without corresponding changes in FM scores. Thus, compensation rather than recovery may underlie the increase in gait speed beyond 6 months post-stroke. Since, at that time, the ma-

jority of individuals neither improve gait speed nor typically receive therapy, future studies should identify interventions and candidates who may benefit from additional gait training.

4.2. Relationship between Change in Gait Speed and Initial Gait Measures

Hypothesis 3 was partially supported because only 9 out of 20 measures at T0 were significantly correlated with the change in gait speed from T0 to T1 (Figure 3). In general, the global gait parameters (speed, stride length/cadence) were inversely associated with the speed change from T0 to T1. The weak correlation with FM-LE suggests that motor impairment within the first 2 months by itself is a poor predictor of the change in gait speed at 6 months post-stroke. This could not be attributed to the variable timing of the initial assessment (T0) because the time from stroke to T0 was not related to the initial FM-LE ($r = -0.20$) or the speed change from T0 to T1 ($r = -0.21$). However, the initial temporal asymmetry of stance and single-support (absolute values) was associated with the later change in gait speed and more so than the initial spatial asymmetry. This reinforces the view that temporal symmetry should be one of the focal points of gait analysis post-stroke [7,8,51–53].

4.3. Study Limitations

Because motor function can recover substantially within the first 2 months in cases of initially mild strokes [38,54,55], some participants might have been close to the plateau in gait recovery by the time of our initial evaluation (e.g., Table 1, #26). Nonetheless, some early good walkers (#40) further improved but they were also younger, which may have played a role. Thus, further studies should combine temporospatial with demographic and clinical predictors of improvement in gait. Caution should be taken when comparing our findings with previous longitudinal studies [16,17,34] because our participants were walking comparably slower at baseline. Like in most previous observational studies, the inability to monitor or retrieve more details about the content of outpatient therapy that the participants reported receiving during the first 3 months after discharge is a limitation of this study. The presented results may have underestimated the potential for gait improvement if the participants did not receive sufficiently intense or focused gait training. However, based on the adopted statistical and MDC criteria, the vast majority achieved prominent gains from baseline to 6 months post-stroke. Finally, some clinically relevant improvements in gait were not captured in our analysis as there were participants who did not change gait speed over time yet they progressed from a walker to a cane, from a quad cane to a single-point cane, or no longer use an assistive device. The whole body gait analysis was not performed to lessen the burden on the participants; however, to fully appreciate the reported temporospatial results, it would be informative to have also limb kinematic data.

5. Conclusions

Among the individuals can who walk independently within 2 months post-stroke, the majority will significantly improve temporospatial gait measures during the first 6 months post-stroke followed by a plateau. Still, at least 20% and up to 40% may continue to increase gait speed from 6 to 12 months post-stroke. Slower ambulators and those with worse temporal symmetry show a greater increase in gait speed from 3–4 weeks to 6 months post-stroke. The improvement in lower-extremity motor function and bilateral improvements in step parameters collectively suggest that gait changes over the first 6 months after stroke reflect a combination of neurological recovery, compensatory strate-gies, and physical therapy received during that time.

Supplementary Materials: The following are available online at https://www.mdpi.com/article/10.3390/brainsci11121648/s1, Table S1: Mean (SD) Fugl-Meyer lower-extremity (FM-LE) motor scores for the paretic leg and temporospatial gait parameters at initial evaluation (T0) and approximately 6 (T1) and 12 months (T2) post-stroke.

Author Contributions: Conceptualization, J.W.C. and D.S.S.; methodology, J.W.C. and D.S.S.; software, J.W.C.; validation, J.W.C. and D.S.S.; formal analysis, J.W.C.; investigation, J.W.C. and D.S.S.; resources, J.W.C. and D.S.S.; data curation, J.W.C.; writing—original draft preparation, J.W.C.; writing—review and editing, J.W.C. and D.S.S.; visualization, J.W.C. and D.S.S.; supervision, J.W.C. and D.S.S.; project administration, J.W.C. and D.S.S.; funding acquisition, D.S.S. All authors have read and agreed to the published version of the manuscript.

Funding: This research was funded in part by the Wilson Research Foundation affiliated with Methodist Rehabilitation Center (Jackson, MS, USA) and the H.F. McCarty, Jr. Family Foundation Fund for Stroke Research.

Institutional Review Board Statement: This study was conducted according to the guidelines of the Declaration of Helsinki, and approved by the Institutional Review Board of Methodist Rehabilitation Center (MRC RP 5009-98, 3 August 2011).

Informed Consent Statement: Informed consent was obtained from all subjects involved in this study.

Data Availability Statement: The data presented in this study are available on request from the corresponding author.

Acknowledgments: We thank Jennifer Sivak and L. Anthony Smith for their assistance in subject recruitment and data acquisition. Chow is the Gertrude C. Ford Director of Motion Analysis.

Conflicts of Interest: The authors declare no conflict of interest.

Appendix A. Determination of Speed Outcome

Because consecutive gait cycles between two sides overlap in time within a walking trial, the average stride velocities over consecutive left/right gait cycles were used to avoid inflating the degrees of freedom of a Welch's unequal variances *t*-test (see the table below, last column). In the case of an unequal number of left and right gait cycles in a walking trial, the outstanding gait cycle (*) was excluded from Welch's *t*-test.

Table A1. An example of stride velocities (m/s) in a walking trial to depict data handling.

Stride #	Left	Right	Average
1	0.653	0.656	0.655
2	0.737	0.745	0.741
3	0.719	0.694	0.706
4	0.646 *	-	-

* Excluded from the Welch's *t*-test.

References

1. Duncan, P.W.; Sullivan, K.J.; Behrman, A.L.; Azen, S.P.; Wu, S.S.; Nadeau, S.E.; Dobkin, B.H.; Rose, D.K.; Tilson, J.K.; Cen, S.; et al. Body-weight-supported treadmill rehabilitation after stroke. *N. Engl. J. Med.* **2011**, *364*, 2026–2036, doi:10.1056/NEJMoa1010790.
2. Katzan, I.; Schuster, A.; Kinzy, T. Physical activity monitoring using a Fitbit device in ischemic stroke patients: Prospective cohort feasibility study. *JMIR Mhealth Uhealth* **2021**, *9*, e14494, doi:10.2196/14494.
3. Kollen, B.; van de Port, I.; Lindeman, E.; Twisk, J.; Kwakkel, G. Predicting improvement in gait after stroke: A longitudinal prospective study. *Stroke* **2005**, *36*, 2676–2680, doi:10.1161/01.STR.0000190839.29234.50.
4. Mizukami, M.; Yoshikawa, K.; Kawamoto, H.; Sano, A.; Koseki, K.; Asakwa, Y.; Iwamoto, K.; Nagata, H.; Tsurushima, H.; Nakai, K.; et al. Gait training of subacute stroke patients using a hybrid assistive limb: A pilot study. *Disabil. Rehabil. Assist. Technol.* **2017**, *12*, 197–204, doi:10.3109/17483107.2015.1129455.
5. Molteni, F.; Guanziroli, E.; Goffredo, M.; Calabrò, R.; Pournajaf, S.; Gaffuri, M.; Gasperini, G.; Filoni, S.; Baratta, S.; Galafate, D.; et al. Gait recovery with an overground powered exoskeleton: A randomized controlled trial on subacute stroke subjects. *Brain Sci.* **2021**, *11*, 104, doi:10.3390/brainsci11010104.
6. Yeung, L.-F.; Lau, C.C.Y.; Lai, C.W.K.; Soo, Y.O.Y.; Chan, M.-L.; Tong, R.K.Y. Effects of wearable ankle robotics for stair and over-ground training on sub-acute stroke: A randomized controlled trial. *J. Neuroeng. Rehabil.* **2021**, *18*, 19, doi:10.1186/s12984-021-00814-6.
7. Brandstater, M.E.; de Bruin, H.; Gowland, C.; Clark, B.M. Hemiplegic gait: Analysis of temporal variables. *Arch. Phys. Med. Rehabil.* **1983**, *64*, 583–587.

8. Chow, J.W.; Stokic, D.S. Gait impairments in patients without lower limb hypertonia early poststroke are related to weakness of paretic knee flexors. *Arch. Phys. Med. Rehabil.* **2019**, *100*, 1091–1101, doi:10.1016/j.apmr.2018.10.014.
9. De Quervain, I.A.; Simon, S.R.; Leurgans, S.; Pease, W.S.; McAllister, D. Gait pattern in the early recovery period after stroke. *J. Bone Joint Surg. Am.* **1996**, *78*, 1506–1514, doi:10.2106/00004623-199610000-00008.
10. Alingh, J.F.; Fleerkotte, B.M.; Groen, B.E.; Rietman, J.S.; Weerdesteyn, V.; van Asseldonk, E.H.F.; Geurts, A.C.H.; Buurke, J.H. Effect of assist-as-needed robotic gait training on the gait pattern post stroke: A randomized controlled trial. *J. Neuroeng. Rehabil.* **2021**, *18*, 1–12, doi:10.1186/s12984-020-00800-4.
11. Ji, S.G.; Kim, M.K. The effects of mirror therapy on the gait of subacute stroke patients: A randomized controlled trial. *Clin. Rehabil.* **2014**, *29*, 348–354, doi:10.1177/0269215514542356.
12. Noh, H.-J.; Lee, S.-H.; Bang, D.-H. Three-dimensional balance training using visual feedback on balance and walking ability in subacute stroke patients: A single-blinded randomized controlled pilot trial. *J. Stroke Cerebrovasc. Dis.* **2019**, *28*, 994–1000, doi:10.1016/j.jstrokecerebrovasdis.2018.12.016.
13. van Bloemendaal, M.; Bus, S.A.; Nollet, F.; Geurts, A.C.H.; Beelen, A. Feasibility and preliminary efficacy of gait training assisted by multichannel functional electrical stimulation in early stroke rehabilitation: A pilot randomized controlled trial. *Neurorehabil. Neural Repair* **2021**, *35*, 131–144, doi:10.1177/1545968320981942.
14. Yang, Y.R.; Yen, J.G.; Wang, R.Y.; Yen, L.L.; Lieu, F.K. Gait outcomes after additional backward walking training in patients with stroke: A randomized controlled trial. *Clin. Rehabil.* **2005**, *19*, 264–273.
15. Aaslund, M.K.; Moe-Nilssen, R.; Gjelsvik, B.B.; Bogen, B.; Næss, H.; Hofstad, H.; Skouen, J.S. A longitudinal study investigating how stroke severity, disability, and physical function the first week post-stroke are associated with walking speed six months post-stroke. *Physiother. Theory Pract.* **2017**, *33*, 932–942, doi:10.1080/09593985.2017.1360424.
16. Berges, I.-M.; Kuo, Y.-F.; Ottenbacher, K.J.; Seale, G.S.; Ostir, G.V. Recovery of functional status after stroke in a tri-ethnic population. *PM&R* **2012**, *4*, 290–295, doi:10.1016/j.pmrj.2012.01.010.
17. Kollen, B.; Kwakkel, G.; Lindeman, E. Hemiplegic gait after stroke: Is measurement of maximum speed required? *Arch. Phys. Med. Rehabil.* **2006**, *87*, 358–363, doi:10.1016/j.apmr.2005.11.007.
18. O'Dell, M.W.; Jaywant, A.; Frantz, M.; Patel, R.; Kwong, E.; Wen, K.; Taub, M.; Campo, M.; Toglia, J. Changes in the activity measure for post-acute care domains in persons with stroke during the first year after discharge from inpatient rehabilitation. *Arch. Phys. Med. Rehabil.* **2021**, *102*, 645–655, doi:10.1016/j.apmr.2020.11.020.
19. Ullberg, T.; Zia, E.; Petersson, J.; Norrving, B. Changes in functional outcome over the first year after stroke: An observational study from the Swedish stroke register. *Stroke* **2015**, *46*, 389–394, doi:10.1161/STROKEAHA.114.006538.
20. Rozanski, G.M.; Wong, J.S.; Inness, E.L.; Patterson, K.K.; Mansfield, A. Longitudinal change in spatiotemporal gait symmetry after discharge from inpatient stroke rehabilitation. *Disabil. Rehabil.* **2020**, *42*, 705–711, doi:10.1080/09638288.2018.1508508.
21. Shin, S.Y.; Lee, R.K.; Spicer, P.; Sulzer, J. Does kinematic gait quality improve with functional gait recovery? A longitudinal pilot study on early post-stroke individuals. *J. Biomech.* **2020**, *105*, 109761, doi:10.1016/j.jbiomech.2020.109761.
22. Kwakkel, G.; Kollen, B.J.; Wagenaar, R.C. Long term effects of intensity of upper and lower limb training after stroke: A randomised trial. *J. Neurol. Neurosurg. Psychiatry* **2002**, *72*, 473–479, doi:10.1136/jnnp.72.4.473.
23. Fugl-Meyer, A.R.; Jääskö, L.; Leyman, I.; Olsson, S.; Steglind, S. The post-stroke hemiplegic patient. 1. a method for evaluation of physical performance. *Scand. J. Rehabil. Med.* **1975**, *7*, 13–31.
24. Sanford, J.; Moreland, J.; Swanson, L.R.; Stratford, P.W.; Gowland, C. Reliability of the Fugl-Meyer assessment for testing motor performance in patients following stroke. *Phys. Ther.* **1993**, *73*, 447–454, doi:10.1093/ptj/73.7.447.
25. Bohannon, R.W.; Smith, M.B. Interrater reliability of a modified Ashworth scale of muscle spasticity. *Phys. Ther.* **1987**, *67*, 206–207, doi:10.1093/ptj/67.2.206.
26. Kadaba, M.P.; Ramakrishnan, H.K.; Wootten, M.E. Measurement of lower extremity kinematics during level walking. *J. Orthop. Res.* **1990**, *8*, 383–392.
27. Stokic, D.S.; Horn, T.S.; Ramshur, J.M.; Chow, J.W. Agreement between temporospatial gait parameters of an electronic walkway and a motion capture system in healthy and chronic stroke populations. *Am. J. Phys. Med. Rehabil.* **2009**, *88*, 437–444, doi:10.1097/PHM.0b013e3181a5b1ec.
28. Zeni, J.A., Jr.; Richards, J.G.; Higginson, J.S. Two simple methods for determining gait events during treadmill and overground walking using kinematic data. *Gait Posture* **2008**, *27*, 710–714.
29. Wang, Y.; Mukaino, M.; Ohtsuka, K.; Otaka, Y.; Tanikawa, H.; Matsuda, F.; Tsuchiyama, K.; Yamada, J.; Saitoh, E. Gait characteristics of post-stroke hemiparetic patients with different walking speeds. *Int. J. Rehabil. Res.* **2020**, *43*, 69–75, doi:10.1097/mrr.0000000000000391.
30. Fagerland, M.W.; Sandvik, L. Performance of five two-sample location tests for skewed distributions with unequal variances. *Contemp. Clin. Trials* **2009**, *30*, 490–496, doi:10.1016/j.cct.2009.06.007.
31. Ruxton, G.D. The unequal variance t-test is an underused alternative to Student's t-test and the Mann-Whitney U test. *Behav. Ecol.* **2006**, *17*, 688–690, doi:10.1093/beheco/ark016.
32. Lewek, M.D.; Sykes, R. Minimal detectable change for gait speed depends on baseline speed in individuals with chronic stroke. *J. Neurol. Phys. Ther.* **2019**, 43, 122–127, doi:10.1097/NPT.0000000000000257.
33. Akoglu, H. User's guide to correlation coefficients. *Turkish J. Emerg. Med.* **2018**, *18*, 91–93, doi:10.1016/j.tjem.2018.08.001.
34. Schober, P.; Schwarte, L.A. Correlation coefficients: Appropriate use and interpretation. *Anesth. Analg.* **2018**, *126*, 1763–1768, doi:10.1213/ANE.0000000000002864.

35. Duncan, P.; Studenski, S.; Richards, L.; Gollub, S.; Lai, S.M.; Reker, D.; Perera, S.; Yates, J.; Koch, V.; Rigler, S.; et al. Randomized clinical trial of therapeutic exercise in subacute stroke. *Stroke* **2003**, *34*, 2173–2180, doi:10.1161/01.STR.0000083699.95351.F2.
36. Bonita, R.; Beaglehole, R. Recovery of motor function after stroke. *Stroke* **1988**, *19*, 1497–1500, doi:10.1161/01.STR.19.12.1497.
37. Branvo, J.P.; Oliveira, S.; Sargento-Freitas, J.; Laíns, J.; Pinheiro, J. Assessing functional recovery in the first six months after acute ischemic stroke: A prospective, observational study. *Eur. J. Phys. Rehabil. Med.* **2019**, *55*, 1–7, doi:10.23736/S1973-9087.18.05161-4.
38. De Wit, L.; Putman, K.; Schuback, B.; Komárek, A.; Angst, F.; Baert, I.; Berman, P.; Bogaerts, K.; Brinkmann, N.; Connell, L.; et al. Motor and functional recovery after stroke: A comparison of 4 European rehabilitation centers. *Stroke* **2007**, *38*, 2101–2107, doi:10.1161/STROKEAHA.107.482869.
39. Jorgensen, H.S.; Nakayama, H.; Raaschou, H.O.; Vive-Larsen, J.; Støier, M.; Olsen, T.S. Outcome and time course of recovery in stroke. Part I: Outcome. The Copenhagen stroke study. *Arch. Phys. Med. Rehabil.* **1995**, *76*, 399–405, doi:10.1016/S0003-9993(95)80567-2.
40. Kwakkel, G.; Kollen, B.; Twisk, J. Impact of time on improvement of outcome after stroke. *Stroke* **2006**, *37*, 2348–2353, doi:10.1161/01.STR.0000238594.91938.1e.
41. Verheyden, G.; Nieuwboer, A.; De, W.L.; Thijs, V.; Dobbelaere, J.; Devos, H.; Severijns, D.; Vanbeveren, S.; De, W.W. Time course of trunk, arm, leg, and functional recovery after ischemic stroke. *Neurorehabil. Neural Repair* **2008**, *22*, 173–179.
42. Bohannon, R.W.; Andrews, A.W.; Glenney, S.S. Minimal clinically important difference for comfortable speed as a measure of gait performance in patients undergoing inpatient rehabilitation after stroke. *J. Phys. Ther. Sci.* **2013**, 25, 1223–1225, doi:10.1589/jpts.25.1223.
43. Fulk, G.D.; Ludwig, M.; Dunning, K.; Golden, S.; Boyne, P.; West, T. Estimating clinically important change in gait speed in people with stroke undergoing outpatient rehabilitation. *J. Neurol. Phys. Ther.* **2011**, *35*, 82–89, doi:10.1097/NPT.0b013e318218e2f2.
44. Geiger, M.; Supiot, A.; Pradon, D.; Do, M.C.; Zory, R.; Roche, N. Minimal detectable change of kinematic and spatiotemporal parameters in patients with chronic stroke across three sessions of gait analysis. *Hum. Mov. Sci.* **2019**, *64*, 101–107, doi:10.1016/j.humov.2019.01.011.
45. Lewek, M.D.; Randall, E.P. Reliability of spatiotemporal asymmetry during overground walking for individuals following chronic stroke. *J. Neurol. Phys. Ther.* **2011**, *35*, 116–121, doi:10.1097/NPT.0b013e318227fe70.
46. Tilson, J.K.; Sullivan, K.J.; Cen, S.Y.; Rose, D.K.; Koradia, C.H.; Azen, S.P.; Duncan, P.W. Locomotor Experience Applied Post Stroke (LEAPS) Investigative Team Meaningful gait speed improvement during the first 60 days poststroke: Minimal clinically important difference. *Phys. Ther.* **2010**, *90*, 196–208, doi:10.2522/ptj.20090079.
47. Beaton, D.E.; Boers, M.; Wells, G.A. Many faces of the minimal clinically important difference (MCID): a literature review and directions for future research. *Curr. Opin. Rheumatol.* **2002**, 14, 109–114, doi:10.1097/00002281-200203000-00006.
48. Copay, A.G.; Subach, B.R.; Glassman, S.D.; Polly, D.W.; Schuler, T.C. Understanding the minimum clinically important difference: a review of concepts and methods. *Spine J.* **2007**, 7, 541–546, doi:10.1016/j.spinee.2007.01.008.
49. Stratford, P.W.; Riddle, D.L. When minimal detectable change exceeds a diagnostic test-based threshold change value for an outcome measure: Resolving the conflict. *Phys. Ther.* **2012**, 92, 1338–1347, doi:10.2522/ptj.20120002.
50. Schmid, A.; Duncan, P.W.; Studenski, S.; Lai, S.M.; Richards, L.; Perera, S.; Wu, S.S. Improvements in speed-based gait classifications are meaningful. *Stroke* **2007**, *38*, 2096–2100, doi:10.1161/STROKEAHA.106.475921.
51. Hsu, A.-L.; Tang, P.-F.; Jan, M.-H. Analysis of impairments influencing gait velocity and asymmetry of hemiplegic patients after mild to moderate stroke. *Arch. Phys. Med. Rehabil.* **2003**, *84*, 1185–1193, doi:10.1016/s0003-9993(03)00030-3.
52. Kim, W.-S.; Choi, H.; Jung, J.-W.; Yoon, J.S.; Jeoung, J.H. Asymmetry and variability should be included in the assessment of gait function in poststroke hemiplegia with independent ambulation during early rehabilitation. *Arch. Phys. Med. Rehabil.* **2021**, *102*, 611–618, doi:10.1016/j.apmr.2020.10.115.
53. Spaich, E.; Svaneborg, N.; Jørgensen, H.R.; Andersen, O. Rehabilitation of the hemiparetic gait by nociceptive withdrawal reflex-based functional electrical therapy: A randomized, single-blinded study. *J. Neuroeng. Rehabil.* **2014**, *11*, 81, doi:10.1186/1743-0003-11-81.
54. Duncan, P.W.; Goldstein, L.B.; Horner, R.D.; Landsman, P.B.; Samsa, G.P.; Matchar, D.B. Similar motor recovery of upper and lower extremities after stroke. *Stroke* **1994**, *25*, 1181–1188, doi:10.1161/01.STR.25.6.1181.
55. Smith, M.-C.; Byblow, W.D.; Barber, P.A.; Stinear, C.M. Proportional recovery from lower limb motor impairment after stroke. *Stroke* **2017**, *48*, 1400–1403, doi:10.1161/STROKEAHA.116.016478.
